# A Synthetic Adjuvant to Enhance and Expand Immune Responses to Influenza Vaccines

**DOI:** 10.1371/journal.pone.0013677

**Published:** 2010-10-27

**Authors:** Rhea N. Coler, Susan L. Baldwin, Narek Shaverdian, Sylvie Bertholet, Steven J. Reed, Vanitha S. Raman, Xiuhua Lu, Joshua DeVos, Kathy Hancock, Jacqueline M. Katz, Thomas S. Vedvick, Malcolm S. Duthie, Christopher H. Clegg, Neal Van Hoeven, Steven G. Reed

**Affiliations:** 1 Infectious Disease Research Institute, Seattle, Washington, United States of America; 2 National Center for Immunization and Respiratory Diseases, Centers for Disease Control and Prevention, Atlanta, Georgia, United States of America; 3 Immune Design Corporation, Seattle, Washington, United States of America; Yale Medical School, United States of America

## Abstract

Safe, effective adjuvants that enhance vaccine potency, including induction of neutralizing Abs against a broad range of variant strains, is an important strategy for the development of seasonal influenza vaccines which can provide optimal protection, even during seasons when available vaccines are not well matched to circulating viruses. We investigated the safety and ability of Glucopyranosyl Lipid Adjuvant-Stable Emulsion (GLA-SE), a synthetic Toll-like receptor (TLR)4 agonist formulation, to adjuvant Fluzone® in mice and non-human primates. The GLA-SE adjuvanted Fluzone vaccine caused no adverse reactions, increased the induction of T helper type 1 (T_H_1)-biased cytokines such as IFNγ, TNF and IL-2, and broadened serological responses against drifted A/H1N1 and A/H3N2 influenza variants. These results suggest that synthetic TLR4 adjuvants can enhance the magnitude and quality of protective immunity induced by influenza vaccines.

## Introduction

Seasonal influenza epidemics are responsible for an average 36,000 deaths and 226,000 hospitalizations annually in the United States alone [1]. Worldwide 3–5 million cases of severe influenza infection occur each year, resulting in between 250,000–500,000 deaths [2]. Although influenza infection rates are highest in children, increased mortality occurs in those older than 65 years, in children less than 2 years of age, and in patients with medical conditions [2].

Vaccination is the best preventative measure against influenza, and acts by increasing neutralizing Abs directed against the HA, the major surface glycoprotein [3]. Currently licensed trivalent seasonal influenza vaccines include antigens from two circulating type A strains and one type B influenza strain. One limitation to seasonal vaccine efficacy is that selected influenza strains are not always representative of the circulating virus, and as a consequence the resulting protection afforded by the vaccine may be reduced [4]. Even for years in which components are antigenically well matched to circulating influenza strains, vaccines are only 60–80% effective at preventing death in older adults aged 65 years and over and are only effective at preventing 70–90% of influenza-specific illnesses in younger adults [2], [4], [5]. When the vaccine is mismatched, efficacy against serologically confirmed influenza illness in adults (18–64 years of age) can be as low as 50% [5]; in those >65 years, efficacy is as low as 35%[4].

Adjuvants provide an important means of improving influenza vaccines. To be effective, these adjuvants should increase the levels of hemagglutination-inhibition (HI) Ab titers against the HA components included in the vaccine and ideally also against variant viruses possessing drifted HA proteins. Emulsion-based adjuvants are currently used in certain licensed seasonal influenza vaccines. MF59 ™ (an oil-in-water adjuvant) combined with a seasonal influenza vaccine, (Fluad®, Novartis Vaccines and Diagnostics) has been approved in some European countries since the late 1990's [6]. In addition, two H5N1 vaccines have recently been approved in Europe: Focetria® (MF59 + H5N1 vaccine; Novartis), and Prepandrix™ (an AS03+H5N1 vaccine; GlaxoSmithKline) [6]. The use of formulations containing oil-in-water emulsions plus Toll-like receptor (TLR) agonists [7] have been successfully used in combination with influenza vaccine and shown to enhance humoral and cellular immunity in animal models compared to using the vaccine alone.

The most advanced TLR4 agonist is an adjuvant derived from the LPS of *Salmonella minnesota* which is known as Monophosphoryl Lipid A (MPL®). MPL® in a variety of formulations has been licensed in Europe as a component of the hepatitis B vaccine Fendrix® [8] and HPV vaccine Cervarix®; both containing MPL/alum. The inclusion of GSK's AS04 Adjuvant System in Cervarix®, recently approved in the US, is believed to significantly broaden immune responses against HPV serotypes not contained in the vaccine.

Expanding on the beneficial effects of MPL®, we have developed a series of formulations containing a synthetic molecule called Glucopyranosyl Lipid A (GLA), which is optimized for binding to the human MD2 molecule of the TLR4 complex. GLA is active on mouse and human antigen presenting cells (APC) ([9]; Coler et al., manuscript submitted), resulting in the induction of cytokines such as IL-12 and TNF.

Here we evaluate the safety and immunogenicity of GLA in a stable-emulsion (GLA-SE) combined with seasonal influenza vaccines in mice and cynomolgus monkeys. The results demonstrate that the adjuvanted Fluzone vaccine had increased humoral and cellular immunogenicity (characterized by a T-helper 1 (T_H_1)-biased immune response). The GLA-SE adjuvanted vaccine caused no adverse side effects in animals and enhanced the potency of the Fluzone vaccine both in terms of Ab and T cell responses and in broadened serological responses against drifted A/H1N1 and an A/H3N2 influenza variant(s) compared to vaccination with Fluzone alone.

## Results

### Safety and Reactogenicity af Fluzone+ GLA-SE

To address the safety of GLA-SE in NHPs, we observed the site of injection for reactogenicity and examined several markers indicative of systemic inflammatory responses. Safety and toxicity data were collected during the course of non-human primate (NHP) dose escalation studies examining the immunogenicity and efficacy of Fluzone+GLA-SE. Two IM administrations of 1 to 50 µg of GLA-SE in combination with Fluzone over 30 days were well tolerated by cynomolgus macaques (Table S1).

There was no evidence of injection site reactions or systemic toxicity in the animals receiving the Fluzone+GLA-SE vaccine or adjuvant alone. No definitive test-article-related changes in hematology were identified. Serum chemistry changes were limited to a mild display of a hepatic acute phase response (mild increases in both CRP and circulating fibrinogen concentrations) after each injection in groups injected with Fluzone+GLA-SE (Table S1).

### The Fluzone+GLA-SE Vaccine Increases and Broadens Hi Ab Levels

We recently reported the immunological effects of combining Fluzone with a research-grade GLA in an oil-in-water emulsion in BALB/c mice [7]. To assess the ability of SE and GLA-SE to enhance Ab activity against influenza strains when formulated with the Fluzone vaccine, we first compared the serum HI titers induced against A/H3N2 influenza variants following immunization in mice. HI titers were measured against the vaccine strain A/Wisconsin/67/2005(H3N2)(Wis/05) and three antigenic variants which represent the A/H3N2 components of past seasonal influenza vaccines: A/Brisbane/10/2007(H3N2), A/Wyoming/02/2003(H3N2) and A/Panama/2007/99(H3N2) (Bris/07, Wyo/03 and Pan/99, respectively). The phylogenetic relationship among all of the A/H3N2 strains except Pan/99 has been recently described [10].

**Figure 1:**
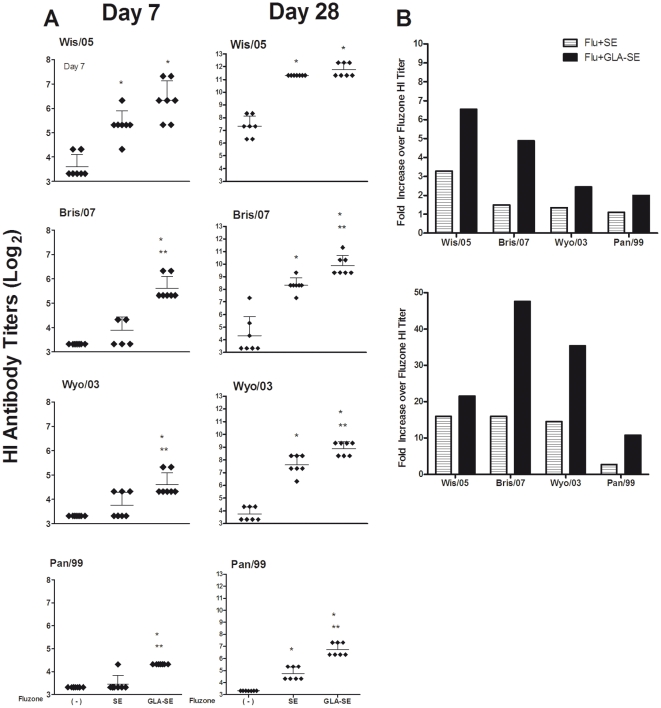
GLA-SE enhances HI titers to the A/H3N2 vaccine component and antigenically drifted A/H3N2 influenza strains. Balb/c mice were immunized with Fluzone (2006–2007) either alone ( - ), or formulated with emulsion (SE), or glucopyranosyl-lipid-adjuvant (GLA-SE). (A) Serum HI titers one week after the prime and boost (N = 7 mice/group). Shown are the individual mouse HI titers (diamonds) and the geometric mean titer (GMT) (indicated by the horizontal bars±the 95% CI) against the vaccine strain (Wis/05) and the antigenically drifted H3N2 strains. The assay detection limit was set at ≤10. Statistical significance between groups was determined and compared to Fluzone (*p<0.05) or Fluzone+SE (**p<0.05). (B) The fold-increase in mean HI titer over that seen with Fluzone is shown for Fluzone+SE or Fluzone+GLA-SE one week after the prime and boost immunizations.

In mice administered a single immunization, only Fluzone formulated with GLA-SE significantly increased HI titers against the vaccine and drifted A/H3N2 strains compared to immunization with Fluzone alone [2 to 6.5-fold increase in titer (p<0.05); Figure 1]. Furthermore, the group given Fluzone+GLA-SE induced significantly higher HI Ab titers against the drifted Bris/07 and Pan/99 strains compared to immunization with Fluzone formulated with SE [(p<0.05); Figure 1]. Following a vaccine boost, Fluzone+SE elicited significantly higher HI titers against the vaccine strain (Wis/05; 16-fold increase in HI titer over Fluzone alone) and the drifted H3N2 strains [(Bris/07,Wyo/03 and Pan/99); 16- and 14-, and 2.7-fold increase in HI titer over Fluzone alone, respectively] [(p<0.05); Figure 1]). The addition of GLA to Fluzone+SE further enhanced HI titers over those achieved either by vaccine alone [21-fold, (p<0.05); Figure 1] or by Fluzone+SE (Figure 1). Fold-increase in HI titers in mice receiving Fluzone+GLA-SE were 47-, 35- and 10.7-fold higher compared with titers elicited by Fluzone alone when tested against the drifted Bris/07, Wyo/03, and Pan/99 strains, respectively [(p<0.05); Figure 1B]. These data demonstrate that combining the trivalent influenza vaccine with either SE or GLA-SE not only enhances vaccine potency, but also induces broader Ab responses against antigenically drifted influenza strains.

**Figure 2:**
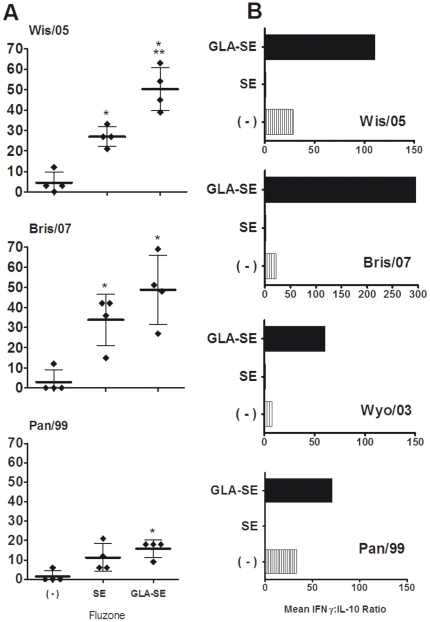
GLA-SE enhances IgG-secreting BMPCs to vaccine and drifted A/H3N2 strains and induces cross-reactive type-1 immunity. Mice were immunized Fluzone (2006–2007) either alone ( - ), or formulated with emulsion (SE), or GLA-SE. (A) Shown are virus-specific [vaccine (Wis/05) or drifted H3N2 strains] IgG-secreting BMPC responses five weeks after boost from individual mice (diamonds; N = 4/group). The group mean is represented by the horizontal bar ± standard deviation (s.d.) per million cells. Statistical significance was determined and compared to Fluzone (*p<0.05) or Fluzone+SE (**p<0.05). (B) IFNγ:IL-10 ratios from immunized mice (N = 4/group) seven weeks after the boost immunization. Splenocytes were harvested and cultured with 5 HAU of inactivated Wis/05, Bris/07, Wyo/03 or Pan/99 influenza virus strains for 3 days. Supernatants were analyzed by ELISA. Results represent the mean IFNγ:IL-10 ratio.

To examine the potential of SE and GLA-SE to induce cells associated with long-lived humoral immunity, we enumerated bone marrow plasma cells (BMPC) that secreted IgG specific for the Wis/05, Bris/07 and Pan/99 influenza strains. When compared to vaccination with Fluzone alone, both the Fluzone+SE and Fluzone+GLA-SE vaccines significantly enhanced virus-specific BMPC levels against these A/H3N2 strains [∼11-fold for the vaccine strain Wis/05, (p<0.001); and ∼16-fold for the drifted Bris/07 strain, (p<0.01), shown in Figure 2A]. Although the Ab levels were lower than for Bris/07 and Wis/05, Fluzone+GLA-SE also enhanced virus-specific BMPC levels against the drifted Pan/99 strain about 10-fold [(p<0.01); Figure 2A]. As expected from the serum IgG results, the GLA-SE-adjuvanted Fluzone vaccine generated significantly more Wis/05-specific BMPC than immunization with Fluzone+SE [∼2 fold; (p<0.01)]. These data indicate that formulating Fluzone with GLA-SE induces a long-lasting humoral response against not only the vaccine strain but also antigenically drifted influenza variants. IFNγ:IL-10 ratios were increased in *in vitro*-stimulated splenocytes collected from mice immunized with GLA-SE and Fluzone compared with those from mice administered Fluzone alone, demonstrating a clear Th1-type immune response (Figure 2B).

Next, we examined whether results observed in mice could be reproduced in a higher animal species. NHPs were immunized with the 2007-08 Fluzone vaccine and geometric mean Ab titers (GMT) against vaccine components [A/Solomon Islands/3/2006(H1N1), A/Wisconsin/67/2005(H3N2), and B/Malaysia/2506/2004] and antigenically drifted strains [A/Brisbane/59/2007(H1N1), A/New Caledonia/20/1999(H1N1), A/Beijing/262/1995(H1N1) and A/Uruguay/716/2007(H3N2)] were measured.

In human clinical studies, the criteria commonly used to assess vaccine immunogenicity include a) rates of seroconversion (a four-fold or greater increase in post-vaccination HI titer compared with pre-vaccination titer) or b) rates of achievement of an HI titer of ≥40. An HI titer of ≥40 is a serologic endpoint generally considered to be associated with at least a 50% reduction in the risk of infection or disease within a population [11]. In this study we measured serum HI titers four weeks after both the prime and boost immunizations. Following a single immunization, the Fluzone vaccine induced HI titers of ≥40 against all 3 components of the vaccine in only 1 of 3 animals (Table S2). The addition of emulsion to the Fluzone vaccine resulted in titers of ≥40 against A/H1N1/Solomon Islands, A/H3N2/Wisconsin, and B/Malaysia components in 2 of 3, 3 of 3, and 1 of 3 animals, respectively. Formulation of Fluzone with GLA-SE (at 25 µg) resulted in titers of ≥40 to the type A components of the vaccine in all 3 animals and against B/Malaysia virus in 2 of 3 animals (Table S2). After the boost, all animals achieved titers of ≥40 titers against all three vaccine components with either Fluzone+SE vaccine or Fluzone+GLA-SE (Table S2). Even after boosting, only 1 of 3 NHP in the Fluzone-alone group achieved titers of  = 40 against the A/H1N1 and B/Malaysia components; these results differ from the stronger responses detected against the A/H3N2 component (Table S2).

**Figure 3:**
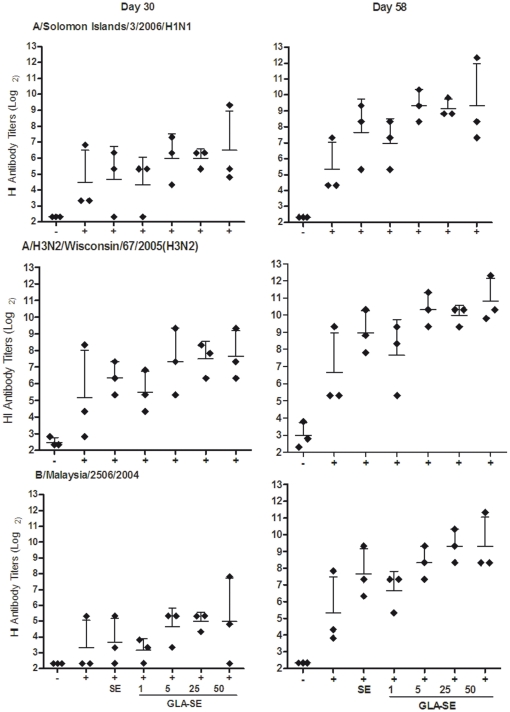
GLA-SE enhances serum HI titers to the A/H1N1 and A/H3N2 components in Fluzone. NHPs (N = 3/group) were injected with saline (-), or were immunized with Fluzone (2007–2008) alone (+), or Fluzone formulated with emulsion (SE), or GLA-SE (1, 5, 25 or 50 µg). Serum samples were collected 4 weeks after the prime and boost immunizations, at day 30 or day 58, respectively. Shown are the HI titers from individual animals (diamonds) and GMT (indicated by the horizontal bars ± the 95% CI) against the three components of the Fluzone vaccine.

In contrast to the observations made in mice, the serum HI Ab titers of NHP following one (day 30) or two immunizations (day 58) with Fluzone+SE were not significantly different compared to the group administered the Fluzone vaccine alone (Figure 3). Nevertheless, following the boost, the inclusion of GLA-SE in the Fluzone vaccine formulations enhanced HI titers in a GLA-SE dose dependent manner. HI titers were increased by, on average, 2–28 fold compared with titers achieved in vaccine groups lacking GLA-SE. After the first immunization, Fluzone formulated with GLA-SE at 5–50 µg doses elicited higher mean serum HI titers to the A/H1N1/Solomon Islands and A/H3N2/Wisconsin components of the vaccine compared with titers elicited by Fluzone alone. Following the boost, animals that received Fluzone+SE, and Fluzone plus GLA-SE, showed a dose-dependent enhancement in the mean serum HI titers to all three components of the vaccine (Figure 3).

A desirable feature of an adjuvanted seasonal influenza vaccine is the ability to induce more broadly cross-reactive Abs against antigenically drifted variants or homosubtypic heterovariant influenza strains that are not included in the vaccine for a given season [12]. To address the ability of SE and GLA-SE to enhance NHP Ab responses against antigenically drifted influenza viruses when formulated with the Fluzone vaccine, we compared the HI titers induced against drifted A/H1N1 and A/H3N2 influenza variants following experimental vaccination.

**Figure 4:**
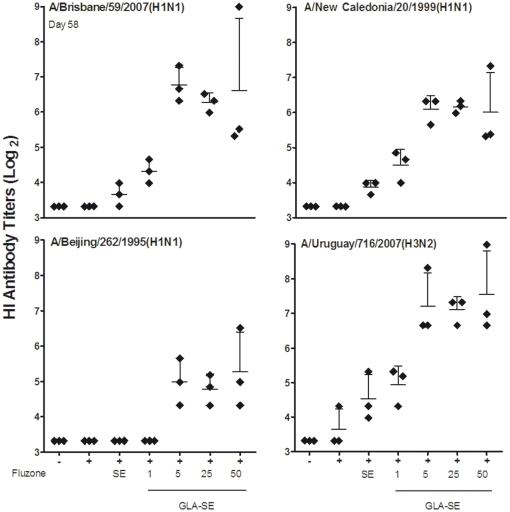
GLA-SE enhances serum HI titers to antigenically drifted A/H1N1 and A/H3N2 influenza strains following a boost immunization. NHPs (N = 3/group) were immunized with Fluzone (2007–2008) alone, or formulated with emulsion (SE), or GLA-SE (1, 5, 25 or 50 µg). Serum samples were collected 4 weeks after the boost (day 58). Shown are the HI titers from individual animals (diamonds) and the GMT (indicated by the horizontal bars ± the 95% CI) against the antigenically A/Brisbane/59/2007(H1N1), A/New Caledonia/20/99(H1N1) and A/Beijing/262/1995(H1N1) strains and the drifted A/Uruguay/716/2007(H3N2) strain.

NHP that were primed and boosted with escalating doses of Fluzone+GLA-SE exhibited higher HI titers not only against the vaccine strain but also to the most recent drifted influenza variant (A/H1N1/Brisbane), a component of the subsequent years vaccine formulation (Figure 4). HI responses against H1N1 strains included in vaccine formulations from 1998 to 2006 were also examined. Vaccination with Fluzone alone did not elicit responses against these strains. However, when Fluzone was administered with 5–50 µg GLA-SE, robust HI titers were achieved (Figure 4) following a boost immunization (cross-reactive titers reached levels of ≥40 to the H1N1/Brisbane and A/H1N1/New Caledonia strains). Titers to the A/H1N1/Beijing strain were also higher in the groups given GLA-SE (5–50 µg) than titers observed with Fluzone, either used alone or combined with SE or the lowest dose of GLA-SE (1 µg), although most of the animals did not achieve titers ≥40 (Figure 4, Table S3).

In addition to testing HI responses to drifted H1N1 strains, ,HI titers were measured against the vaccine strain (A/H3N2/Wisconsin) (Figure 3) and one antigenically drifted A/H3N2 strain included in seasonal influenza vaccines in 2008 (A/H3N2/Uruguay) (Figure 4). Although Fluzone+SE stimulated higher HI titers to the vaccine strain than Fluzone alone, a boost was required to induce the higher titers to the drifted A/H3N2/Uruguay strain (Figure 4). All of the NHPs that received Fluzone+GLA-SE (5–50 µg) exhibited cross-reactive HI titers well above 40 against the drifted H3N2 strain following two immunizations, and 2 of 3 NHPs given the lowest dose GLA-SE (1 µg) achieved this titer. In contrast, only 1 of 3 NHPs reached a cross reactive titer ≥40 following immunization with Fluzone+SE, and none of the animals given Fluzone without adjuvant responded to the drifted strain (Figure 4, Table S3).

### Cytokine Response in Immunized Animals

Cellular immune responses, including T_H_1-type responses, play a critical role in the clearance of influenza viruses in animal models and are associated with protection against influenza-related illness in the elderly. We, therefore, assessed the ability of the GLA-SE- and SE-formulated Fluzone vaccines to elicit influenza-specific cellular responses. In initial studies, spleen cells from mice immunized with Fluzone alone or with either SE or GLA-SE were restimulated *in vitro* with the vaccine strain or with the antigenically drifted A/H3N2 influenza The levels of cytokine produced in response to stimulation by any A/H3N2 strain were comparable, suggesting that the cellular responses are most likely directed towards conserved proteins within the antigen mixture (Figure S1 (7)). Fluzone+GLA-SE vaccinated mice induced significantly greater levels of antigen-specific IFNγ compared to responses from mice immunized with Fluzone+SE or Fluzone alone (Figure S1 (7)). In sharp contrast to GLA-SE, the vaccine formulated with SE induced a strong virus-specific T_H_2 cytokine response characterized by significantly greater levels of IL-5 compared to Fluzone alone or Fluzone+GLA-SE (Figure S1 (7)). Greater levels of IL-10 were induced in response to the vaccine with SE compared to GLA-SE (Figure S1 (7)). Comparison of IFNγ to IL-10 levels, measured after the second immunization as shown in Figure 2B, shows that GLA-SE directs type-1 cellular responses, characterized by the production of IFNγ. These data suggest that the virus-specific recall responses can be modulated by formulating Fluzone; inclusion of an oil-in-water emulsion induces a T_H_2 response whereas the addition of a TLR4 agonist to the emulsion directs a T_H_1 response.

**Figure 5:**
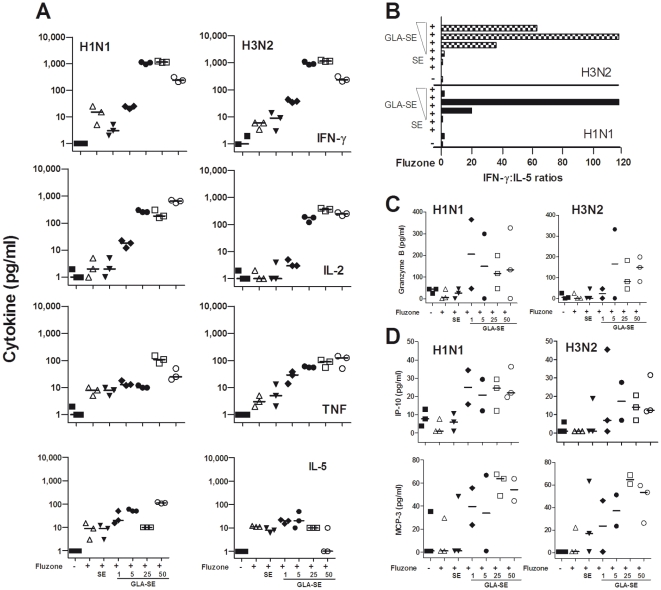
Cellular responses after injection with saline, Fluzone alone, or combined with SE or GLA-SE. NHP (N = 3/group) were given 2 injections, 4 weeks apart with saline, Fluzone, Fluzone+SE or Fluzone+GLA-SE (1, 5, 25 and 50 µg). (A) IFNγ, IL-2, TNF and IL-5 (pg/mL) recall responses after stimulation of whole blood with H1N1 or H3N2 were evaluated by CBA at day 0 and day 58. (B) IFNγ:IL-5 ratios of cytokine recall responses after stimulation of whole blood with H1N1 or H3N2, were evaluated by CBA at day 58. (C) Granzyme B levels (pg/mL) responses in whole blood following stimulation with H1N1 or H3N2 was evaluated by Luminex at day 58. Horizontal lines represent median Granzyme B concentrations. (D) Chemokine responses to Fluzone antigens after immunization. MCP-3 and IP-10 (pg/mL) responses in whole blood following stimulation with H1N1 or H3N2 were evaluated by Luminex at day 58. Horizontal lines represent median chemokine concentrations.

In additional studies we used whole blood assays (WBA) to assess antigen-specific cytokine responses of immunized and non-immunized monkeys (Figure 5A). To evaluate T cell responsiveness to the H1N1 and H3N2 components of the Fluzone vaccine, the levels of IFNγ, TNF, IL-2, IL-6, IL-5 and IL-4 secreted during WBA were evaluated by a T_H_1/T_H_2 Cytokine Bead Array. All of the NHPs immunized with any of the Fluzone+GLA-SE (5-25 µg) vaccines produced increased H1N1- and H3N2-specific IFNγ responses four weeks after the second immunization compared to NHPs that were immunized with the Fluzone alone, Fluzone with SE, or Fluzone with 1 µg GLA-SE (Figure 5A). All animals that received the booster immunizations with Fluzone+GLA-SE at 25 and 50 µg also had increased IL-2 and TNF levels compared to those receiving Fluzone alone, Fluzone+SE, or Fluzone+GLA-SE (1 or 5 µg) boosts (Figure 5A). Thus, only animals receiving the Fluzone+GLA-SE at the highest doses (25 or 50 µg GLA) produced elevated levels of all three of the T_H_1-associated cytokines IFNγ, TNF and IL-2. The cytokine responses 4 weeks post-boost (day 58) revealed that NHP boosted with Fluzone alone and Fluzone+SE had specific H1N1 and H3N2 responses marked by lower T_H_1:T_H_2 cytokine ratios than those immunized with Fluzone+GLA-SE at 5, 25 and 50 µg (Figure 5B). The highest IFNγ:IL-5 ratios (>100) produced in response to both the H3N2 and H1N1 components of the Fluzone vaccine were observed in animals immunized with Fluzone +25 µg of GLA-SE. Interestingly, the addition of GLA-SE to the Fluzone vaccine increased median levels of H1N1- and H3N2-specific Granzyme B (Figure 5C), as well as several chemokines. These chemokines [(C-X-C motif) ligand 10 (CXCL10), also called IP-10) and monocyte-chemotactic-protein-3 (MCP-3; also called chemokine (C-C motif) ligand 7 (CCL7)] are selectively expressed on monocytes, endothelial cells, and fibroblasts, and all are chemotactic for and activate a great variety of inflammatory cell types including monocytes/macrophages, T cells, NK cells, and/or dendritic cells (Figure 5D).

## Discussion

Next generation influenza vaccines that potentiate immune responses and elicit protection against a broader range of antigenically drifted strains are urgently needed. To further advance development of such vaccines, we have analyzed the impact on immune responses of incorporating a stable oil-in-water emulsion and a synthetic TLR4 agonist into a standard influenza vaccine. The experiments described in this report are the first to evaluate an oil-in-water emulsion (SE) with various concentrations of this synthetic TLR4 agonist (GLA), all in combination with Fluzone, a licensed trivalent inactivated influenza vaccine for its safety and ability to significantly enhance immune responses in both mice and NHP. With the ever increasing need for safe ways to either improve vaccine efficacy and/or to provide dose sparing effects, most candidate adjuvants fail to reach the market place because of safety issues including toxicity or reactogenicity particularly at the site of injection. Though increased levels of pro-inflammatory cytokines were observed in the context of GLA-SE, there was no evidence of injection site reactions or systemic toxicity in the animals receiving the Fluzone+GLA-SE vaccine or adjuvant alone.

Our results demonstrate that incorporating SE into Fluzone enhances Ab responses. Furthermore, the incorporation of GLA with SE enhances the same Ab responses, stimulates production of Abs that cross-react with antigenically variant influenza strains not present in the vaccine, and generates T_H_1 cellular immune responses. Thus, our studies clearly demonstrate that GLA-SE provides further enhancement and broadening of the immune responses already elevated by the addition of SE to Fluzone.

The ability of SE to adjuvant flu vaccines, as observed in our studies, is in general agreement with those previously reported for another oil-in-water adjuvant, MF59™ [13], [14]. After two immunizations, the addition of SE to the Fluzone vaccine increased HI titers to the A/H1N1/Solomon Islands and the A/H3N2/Wisconsin components of the Fluzone vaccine to seroprotective levels HI titers of ≥40. In contrast to previous reports indicating the generation of cross-reactive Abs against heterovariant influenza strains by the addition of oil-in-water adjuvants [14], our results failed to demonstrate that the addition of SE alone to Fluzone could increase HI titers against antigenically drifted influenza variants. It should be noted that we used a low dose of Fluzone vaccine and that even when administered with SE, this may not be a potent enough combination to stimulate cross-reactive antigen-specific responses. Regardless, our results confirm those of others which indicate that incorporation of SE in influenza vaccines is an important step in the development of more effective seasonal and pandemic influenza vaccines.

Our results clearly demonstrate that, compared to vaccination with Fluzone alone or Fluzone formulated with SE, the further addition of GLA increases cross-protective HI Ab responses against antigenic variants. In this study, a single administration of Fluzone with GLA-SE leads to the generation of HI titers at seroprotective levels in a majority of NHP. GLA-SE further enhanced HI titers in NHP following two immunizations, as compared to either SE plus Fluzone or Fluzone alone. Previous studies have shown that other TLR agonists, such as the TLR9 agonist CpG, enhance cellular interactions within germinal centers, leading to the selection of B cells with a high affinity for antigen binding [15]. GLA-SE broadened HI responses and generated more virus-specific BMPCs, the hallmark of long-term humoral immunity [16], compared with SE alone. Taken together, these results suggest that a GLA-SE-adjuvanted influenza vaccine may elicit not only more durable but also more broadly reactive Abs that have the potential to extend the protective lifespan of the vaccine.

Our results also show that the addition of GLA-SE to the Fluzone vaccine selectively shifts the cellular immune response toward a type-1 cellular immune response pattern, characterized by the production of IFNγ. TLR4 agonists, such as MPL, have been shown to be potent adjuvants in a range of species, including humans [8], [17]. NHP immunized with GLA-SE-adjuvanted Fluzone vaccine produced higher Ab titers and type-1 cytokine levels than those found in animals immunized with Fluzone alone or a Fluzone+SE formulation. As many aging individuals experience a shift from T_H_1 to T_H_2 responses [3] and a concomitant increase in susceptibility to influenza disease, a potent anti-influenza cellular response may be of particular importance in older adults. Many elderly patients respond poorly to the existing influenza vaccines [18], [19]. The ability of the GLA-SE adjuvant to induce potent type-1 cellular immune responses is in marked contrast to the T_H_2 response induced by traditional adjuvants such as Alum and MF59 ™ [20]. The anti-viral activity of type-1 cytokines such IFNγ and TNF [21], [22], [23], and the synergistic effect of these cytokines, against human influenza viruses is well known [24]. The enhanced induction of both IFNγ and TNF by Fluzone adjuvanted with GLA-SE could positively impact an influenza vaccination program aimed at the elderly population since prior studies have reported a putative correlation of cell mediated immunity and protection against influenza.

The weakened immune system of elderly people presents particular challenges in the development of vaccines and adjuvants for this population. There are well recognized changes in the function of APCs, T cells and B cells in older people. Several strategies to improve vaccination in the elderly have been explored. A number of studies have shown that the use of adjuvants (e.g. emulsions and TLR agonists) could trigger more potent activation of APCs. Like other TLR4 containing adjuvants, we have preliminary data demonstrating that GLA can induce robust immune responses in aged mice and humans whose immune systems have undergone significant decline with increasing age. Recent studies suggest that T cell responses, rather than Ab, may be a better immune correlate of protection against influenza in older adults but that this population overall may have impaired T_H_1 responses [3], [19].

The GLA-SE-adjuvanted Fluzone vaccine described in this study elicits high IFNγ:IL-10 and IFNγ:IL-5 ratios that clearly show a dominant type-1 cellular immune response. Potent virus-specific IFNγ responses are also thought to be capable of reversing the age-related impairments of CTL activity considered essential for viral clearance [25]. Therefore, the robust type-1 cellular immune response elicited by GLA-SE may have implications for the development of influenza vaccines better suited for the elderly. Conversely, the oil-in-water-based adjuvant we evaluated clearly shifted responses towards a type-2 cellular immune response, dominated by IL-5 (and IL-10), results consistent with other studies evaluating oil-in-water-based adjuvants, including MF59™ [26]. We show that, in the absence of the TLR4 agonist, the oil-in-water emulsion adjuvant potentiates T_H_2 responses reported to adversely affect viral clearance in animal models [27]. Thus, the data described herein suggests that influenza vaccines combined with GLA-SE could provide an additional benefit to the oil-in-water emulsion adjuvants by generating more broadly cross-reactive Ab responses, and by potentiation of T_H_1 responses.

## Materials and Methods

### Animals

Female BALB/c mice 6–7 weeks (Charles River Laboratories, Wilmington, MA) were maintained under SPF conditions by IDRI Animal Care and Use Committee (IACUC).

Twenty-one confirmed healthy male Cynomologus macaques ages 4.7 to 6.6 years old and weights of 4.81 to 6.47 kg were housed at SNBL USA (Everett, WA) in accordance with SNBL USA's IACUC. Monkeys were pre-screened for their influenza status, randomized by body weights and assigned into study groups.

### Immunizations

The 2006–2007 Fluzone influenza vaccine formulated for the Northern Hemisphere (Sanofi Pasteur, Swiftwater, PA), containing antigens from A/New Caledonia/20/99(H1N1), A/Wisconsin/67/2005(H3N2) and B/Malaysia/2506/2004 was used for the murine studies. The 2007–2008 Northern Hemisphere Fluzone influenza vaccine containing antigens from A/Solomon Islands/3/2006(H1N1), A/Wisconsin/67/2005(H3N2) and B/Malaysia/2506/2004, was used for the NHP studies. Fluzone was combined with either IDRI's oil-in-water emulsion (SE) or SE containing a synthetic TLR4 agonist (GLA-SE), before administration to animals. Adjuvant formulations were prepared as previously described [28].

Mice were immunized IM twice, 3 weeks apart. Mice received a low dose (0.2 µg total HA) of the 2006–2007 Fluzone vaccine with or without SE, or GLA-SE (20 µg).

NHP (N = 3/group) were immunized by IM injection on days 1 and 30 with the pediatric dose, which included 7.5 µg HA/strain resulting in a total HA dose of 22.5 µg, of the 2007–2008 Fluzone vaccine alone, or adjuvanted with SE, or escalating doses (1, 5, 25, or 50 µg) of GLA-SE.

### Influenza Strains and Proteins used for murine HI and cytokine assays

To evaluate the ability of the vaccines to induce serum Ab responses against heterovariant influenza strains, vaccine and non-vaccine drifted influenza A strains were selected for use in HI assays and splenocyte cultures. A/Wisconsin/67/2005(H3N2)(Wis/05), A/Wyoming/03/2003(H3N2)(Wyo/03) and A/Panama/2007/99(H3N2)(Pan/99) strains were provided by the CDC and Prevention (Atlanta, GA) and purified viral protein from A/Brisbane/10/2007(H3N2)(Bris/07) was purchased from ProSpec (Rehovot, Israel). All of the viruses used were grown in the allantoic cavity of embryonated eggs and were stored at −80°C until use.

### Clinical Assessments of NHP

Injection sites were evaluated prior to each inoculation, at 2 hours ±30 minutes and again at 24, 48, 72, 96, 120 and 144±1 hour after each dose, using a Draize dermal irritation scoring system [29]. Clinical observations to establish the general safety of vaccine administration were collected throughout the investigation. Limb use impairment and food consumption were evaluated for each monkey. The following parameters were also measured: (1) body weight (weekly throughout the study), (2) rectal temperature (at intervals prior to and following each dose, and at 6, 12 and 24 hours±30 minutes after each dose), (3) urinalysis, (4) hematological parameters, (5) coagulation, and (6) serum chemistry (shown in Table S1)). Blood samples were collected 5 days before, on the day of, and 2, 30 and 58 days after the initial inoculation. Serum was stored at −60°C until analysis.

### Influenza strains used for NHP HI assays

For the NHP HI assays, the 2007 vaccine components, including A/Solomon Islands/3/2006(H1N1), A/Wisconsin/67/2005(H3N2) and B/Malaysia/2506/2004 plus all drifted A (H1N1) strains incorporated in seasonal influenza vaccines since 1998 including A/Brisbane/59/2007(H1N1), A/New Caledonia/20/99(H1N1) and A/Beijing/262/1995(H1N1) and the drifted A/Uruguay/716/2007(H3N2) influenza strains were evaluated. All viruses were provided by the Centers for Disease Control and Prevention (Atlanta, GA).

### Bone Marrow ELISPOT

Antigen-specific mouse bone marrow plasma cells were quantified by ELISPOT as previously described, five weeks after the second immunization (7). Purified and concentrated A/Wisconsin/67/2005(H3N2)(Wis/05), A/Brisbane/10/2007(H3N2)(Bris/07) and A/Panama/2007/99(H3N2)(Pan/99) viral antigens were purchased from ProSpec for use in the ELISPOT.

### Cellular Immune Responses

Seven weeks after the second immunization, mice were euthanized and spleens (N = 4/group) were harvested. Splenocytes were cultured with 5 HAU of inactivated A/Wisconsin/67/2005(H3N2)(Wis/05), A/Brisbane/10/2007(H3N2)(Bris/07), A/Wyoming/03/2003(H3N2)(Wyo/03) or A/Panama/2007/99(H3N2)(Pan/99) influenza virus at 37°C in 5% CO2 for 72 hrs following a method adapted from Lu et al [30]. Culture supernatants were then collected and cytokine levels were measured by ELISA according to the manufacturer's instructions (IFNã and IL-10, eBioscience, San Diego, CA).

Monkeys were bled at day 0 and 58 to evaluate cellular immune responses by whole blood assay (WBA). Heparinized blood (450 µL) was dispensed into 48-well plates (ICN Biomedicals, Inc). Phytohemagglutinin (PHA; 100 µg/mL), Fluzone (9 µg/mL), purified rH3 from A/Wisconsin/67/2005(H3N2) (10 µg/mL) (Protein Sciences Corp., Meriden, CT), inactivated A/Solomon Islands/3/2006(H1N1) virus (10HAU/mL) (provided by the Centers for Disease Control and Prevention) or saline was then added to provide stimulation in a final volume of 500 µL/well in duplicate. Following incubation at 37°C, 5% CO_2_ for 24 hours, 200 µL plasma was collected and IFNγ,? TNF, IL-2, IL-6, IL-5, IL-4, Granzyme B, MCP3 and IP-10 levels measured using a T_H_1/T_H_2 cytometric bead array (CBA) kit (Becton Dickinson) or a custom Luminex panel (Panomics).

### HI Assay

Sera were collected from mice one week after each immunization. Anti-HI Abs specific to the A/H3N2 component of the 2006–2007 Northern Hemisphere Fluzone formulation, A/Wisconsin/67/2005(H3N2)(Wis/05), and to the A/H3N2 component of other seasonal influenza vaccines used since 2001 including A/Brisbane/10/2007(H3N2)(Bris/07), A/Wyoming/03/2003(H3N2)(Wyo/03) or A/Panama/2007/99(H3N2)(Pan/99) were determined as previously described using 0.5% turkey RBC [31]. Mouse sera were treated with receptor destroying enzyme followed by heat treatment (56°C, 30 min) before use in the HI assay. The HI titer was defined as the reciprocal of the highest dilution of sera which completely inhibits the agglutination of the turkey RBCs.

Sera were collected from NHP on Day 1, 30 and 58 and evaluated for the presence of hemagglutination inhibiting antibodies specific to the 2007–2008 Northern Hemisphere Fluzone components including A/Solomon Islands/3/2006(H1N1), A/Wisconsin/67/2005(H3N2) and B/Malaysia/2506/2004 and also against drifted A (H1N1) strains incorporated in seasonal influenza vaccines since 1998 including A/Brisbane/59/2007(H1N1), A/New Caledonia/20/99(H1N1) and A/Beijing/262/1995(H1N1) and the drifted A/Uruguay/716/2007(H3N2) influenza strain by means of established methods, using four hemaglutinating units (HAU) of virus and 0.5% turkey RBCs). NHP sera were treated with receptor destroying enzyme followed by heat treatment (56°C, 30 min) before use in the HI assay. Three separate HI assays were conducted on each serum sample and each individual serum sample was assayed in duplicate in each assay. The seroprotective rate was defined as the percentage of animals per vaccine group that achieved a post-vaccination HI titer of ≥ 40 HI. Preimmune titers in all animals were ≤10.

### Statistical Analysis

Statistical analysis was performed by a standard one-way ANOVA with the Tukey Multiple Comparison Test between the groups using GraphPad Prism (GraphPad Software, San Diego, CA).

## Supporting Information

Figure S1 Virus-specific cytokine responses 7 weeks post boost with the 2006-2007 Fluzone influenza vaccine alone (-) or formulated with either an oil and water emulsion (SE) or a synthetic TLR4 agonist formulation (GLA-SE). Splenocytes were harvested from 4 mice/vaccine group and cultured with 5 HAU of inactived Wis/05, Bris/07, Wyo/03 or Pan/99 influenza strains. Supernatants were collected 72 hours post culture and analyzed by ELISA. Values represent the average virus-specific cytokine responses detected in cultures +SD. One asterisk represents significantly greater responses to Fluzone alone (p<0.05). Two asterisks represents significantly greater responses to Fluzone+SE (p<0.05). Three asterisks represents significantly greater responses to Fluzone +GLA-SE (p<0.05).
(0.06 MB PDF)

Table S1 (0.05 MB DOC)

Table S2 (0.04 MB DOC)

Table S3 (0.04 MB DOC)

## References

[1] Katz JM, Veguilla V, Belser JA, Maines TR, Van Hoeven N (2009). The public health impact of avian influenza viruses.. Poult Sci.

[2] WHO (2009). WHO Fact Sheet No. 211. Fact Sheet No. 211, April 2009.

[3] McElhaney JE, Xie D, Hager WD, Barry MB, Wang Y (2006). T cell responses are better correlates of vaccine protection in the elderly.. J Immunol.

[4] Carrat F, Flahault A (2007). Influenza vaccine: the challenge of antigenic drift.. Vaccine.

[5] Bridges CB, Thompson WW, Meltzer MI, Reeve GR, Talamonti WJ (2000). Effectiveness and cost-benefit of influenza vaccination of healthy working adults: A randomized controlled trial.. JAMA.

[6] Vogel FR, Caillet C, Kusters IC, Haensler J (2009). Emulsion-based adjuvants for influenza vaccines.. Expert Rev Vaccines.

[7] Baldwin SL, Shaverdian N, Goto Y, Duthie MS, Raman VS (2009). Enhanced humoral and Type 1 cellular immune responses with Fluzone adjuvanted with a synthetic TLR4 agonist formulated in an emulsion.. Vaccine.

[8] Alderson MR, McGowan P, Baldridge JR, Probst P (2006). TLR4 agonists as immunomodulatory agents.. J Endotoxin Res.

[9] Baldwin SL, Bertholet S, Kahn M, Zharkikh I, Ireton GC (2009). Intradermal immunization improves protective efficacy of a novel TB vaccine candidate.. Vaccine.

[10] Barr IG, McCauley J, Cox N, Daniels R, Engelhardt OG (2010). Epidemiological, antigenic and genetic characteristics of seasonal influenza A(H1N1), A(H3N2) and B influenza viruses: basis for the WHO recommendation on the composition of influenza vaccines for use in the 2009-2010 Northern Hemisphere season.. Vaccine.

[11] de Jong JC, Palache AM, Beyer WE, Rimmelzwaan GF, Boon AC (2003). Haemagglutination-inhibiting antibody to influenza virus.. Dev Biol (Basel).

[12] de Jong JC, Beyer WE, Palache AM, Rimmelzwaan GF, Osterhaus AD (2000). Mismatch between the 1997/1998 influenza vaccine and the major epidemic A(H3N2) virus strain as the cause of an inadequate vaccine-induced antibody response to this strain in the elderly.. J Med Virol.

[13] Wack A, Baudner BC, Hilbert AK, Manini I, Nuti S (2008). Combination adjuvants for the induction of potent, long-lasting antibody and T-cell responses to influenza vaccine in mice.. Vaccine.

[14] Banzhoff A, Nacci P, Podda A (2003). A new MF59-adjuvanted influenza vaccine enhances the immune response in the elderly with chronic diseases: results from an immunogenicity meta-analysis.. Gerontology.

[15] Pasare C, Medzhitov R (2005). Control of B-cell responses by Toll-like receptors.. Nature.

[16] Slifka MK, Matloubian M, Ahmed R (1995). Bone marrow is a major site of long-term antibody production after acute viral infection.. J Virol.

[17] Stoute JA, Heppner DG, Mason CJ, Siangla J, Opollo MO (2006). Phase 1 safety and immunogenicity trial of malaria vaccine RTS,S/AS02A in adults in a hyperendemic region of western Kenya.. Am J Trop Med Hyg.

[18] Weksler ME, Szabo P (2000). The effect of age on the B-cell repertoire.. J Clin Immunol.

[19] Deng Y, Jing Y, Campbell AE, Gravenstein S (2004). Age-related impaired type 1 T cell responses to influenza: reduced activation ex vivo, decreased expansion in CTL culture in vitro, and blunted response to influenza vaccination in vivo in the elderly.. J Immunol.

[20] Baudner BC, Ronconi V, Casini D, Tortoli M, Kazzaz J (2009). MF59 emulsion is an effective delivery system for a synthetic TLR4 agonist (E6020).. Pharm Res.

[21] Baumgarth N, Kelso A (1996). In vivo blockade of gamma interferon affects the influenza virus-induced humoral and the local cellular immune response in lung tissue.. J Virol.

[22] Belz GT, Wodarz D, Diaz G, Nowak MA, Doherty PC (2002). Compromised influenza virus-specific CD8(+)-T-cell memory in CD4(+)-T-cell-deficient mice.. J Virol.

[23] Brown DM, Roman E, Swain SL (2004). CD4 T cell responses to influenza infection.. Semin Immunol.

[24] Wong GH, Goeddel DV (1986). Tumour necrosis factors alpha and beta inhibit virus replication and synergize with interferons.. Nature.

[25] Mbawuike IN, Acuna C, Caballero D, Pham-Nguyen K, Gilbert B (1996). Reversal of age-related deficient influenza virus-specific CTL responses and IFN-gamma production by monophosphoryl lipid A.. Cell Immunol.

[26] Singh M, Carlson JR, Briones M, Ugozzoli M, Kazzaz J (1998). A comparison of biodegradable microparticles and MF59 as systemic adjuvants for recombinant gD from HSV-2.. Vaccine.

[27] Doherty PC, Topham DJ, Tripp RA, Cardin RD, Brooks JW (1997). Effector CD4+ and CD8+ T-cell mechanisms in the control of respiratory virus infections.. Immunol Rev.

[28] Anderson RC, Fox CB, Dutill TS, Shaverdian N, Evers TL (2010). Physicochemical characterization and biological activity of synthetic TLR4 agonist formulations.. Colloids Surf B Biointerfaces.

[29] Draize JH (1959). Appraisal of the Safety of Chemicals in Foods, Drug and Cosmetics..

[30] Lu X, Clements JD, Katz JM (2002). Mutant Escherichia coli heat-labile enterotoxin [LT(R192G)] enhances protective humoral and cellular immune responses to orally administered inactivated influenza vaccine.. Vaccine.

[31] Kendal AP SJ, Pereira MS (1982). Concepts and procedures for laboratory-based influenza surveillance..

