# Supplementary material for: A Synthetic Adjuvant to Enhance and Expand Immune Responses to Influenza Vaccines
Source: PLoS One. 2010 Oct 27;5(10):e13677. doi: 10.1371/journal.pone.0013677 (PMC2965144; doi:10.1371/journal.pone.0013677)
Supplement: Figure S1 — Virus-specific cytokine responses 7 weeks post boost with the 2006-2007 Fluzone influenza vaccine alone (-) or formulated with either an oil and water emulsion (SE) or a synthetic TLR4 agonist formulation (GLA-SE). Splenocytes were harvested from 4 mice/vaccine group and cultured with 5 HAU of inactived Wis/05, Bris/07, Wyo/03 or Pan/99 influenza strains. Supernatants were collected 72 hours post culture and analyzed by ELISA. Values represent the average virus-specific cytokine responses detected in cultures +SD. One asterisk represents significantly greater responses to Fluzone alone (p<0.05). Two asterisks represents significantly greater responses to Fluzone+SE (p<0.05). Three asterisks represents significantly greater responses to Fluzone +GLA-SE (p<0.05). (0.06 MB PDF) [file pone.0013677.s001.pdf]

Supplemental Figure S1

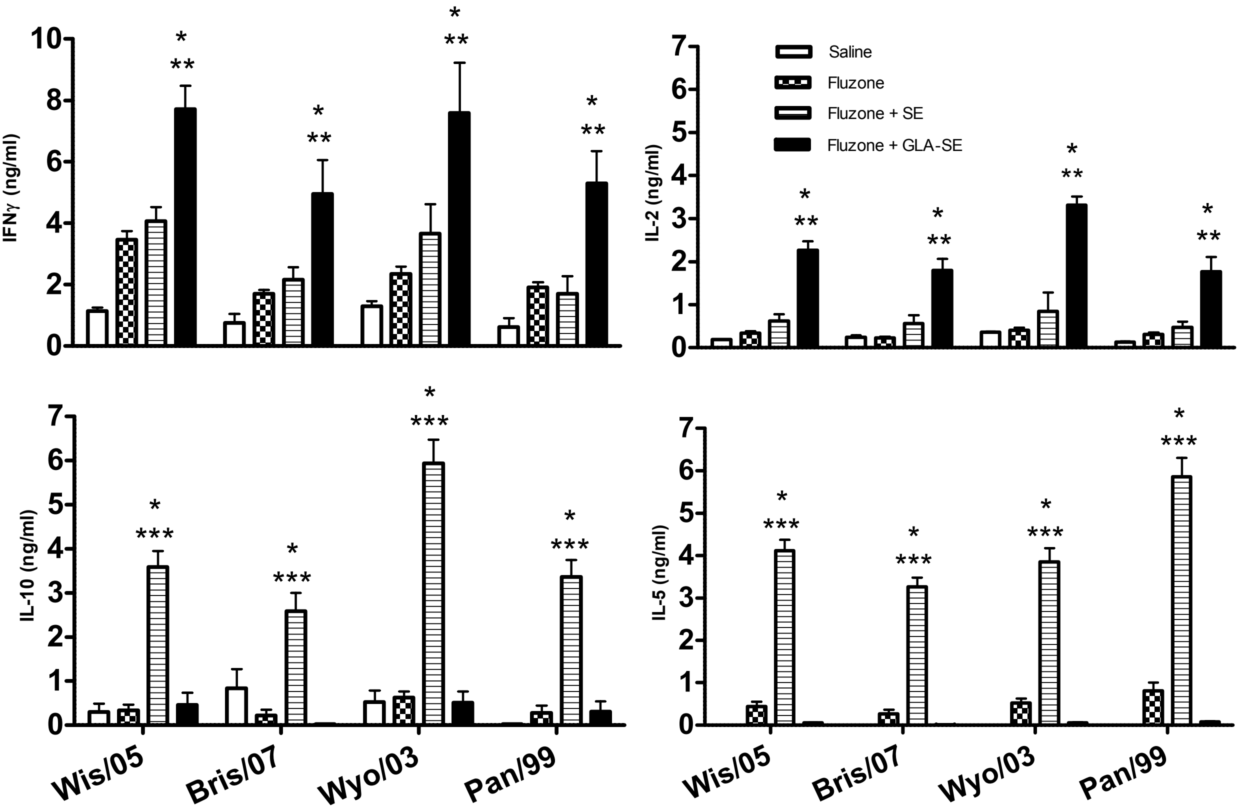

Virus specific cytokine responses 7 weeks post boost with the 2006-2007 Fluzone influenza vaccine alone (-) or formualted with either an oil and water emulsion (SE) or a synthetic TLR4 agonist formulation (GLA-SE). Splenocytes were harvested from 4 mice/vaccine and cultured with 5 HAU of inactived Wis/05, Bris/07, Wyo/03 or Pan/99 influenza strains. Supernatants were collected 72hrs post culture and analyzed by ELISA. Values represent the average virus specific cytokine responses detected in cultures +SD. One asterisk represents significantly greater responses to Fluzone alone ($p<0.05$). Two asterisks represents significantly greater responses to Fluzone+SE ($p<0.05$). Three asterisks represent signifigantly geater responses to Fluzone +GLA-SE ($p<0.05$).
