# Supplementary material for: A Synthetic Adjuvant to Enhance and Expand Immune Responses to Influenza Vaccines
Source: PLoS One. 2010 Oct 27;5(10):e13677. doi: 10.1371/journal.pone.0013677 (PMC2965144; doi:10.1371/journal.pone.0013677)
Supplement: Table S1 — (0.05 MB DOC) [file pone.0013677.s002.doc]

**Supplemental Table 1.** Safety of Adjuvanted Fluzone Vaccines in NHP.

|  | Vaccines | | | | | | |
| --- | --- | --- | --- | --- | --- | --- | --- |
| In life monitoring | Saline | Fluzone | Fluzone +SE | Fluzone +GLA-SE (1µg) | Fluzone+GLA-SE (5µg) | Fluzone+GLA-SE (25µg) | Fluzone +GLA-SE (50µg) |
| AE | ^a^0/3 | 0/3 | 0/3 | 0/3 | 0/3 | 0/3 | 0/3 |
| Serious AE | 0/3 | 0/3 | 0/3 | 0/3 | 0/3 | 0/3 | 0/3 |
| ^b^Induration | 0/3 | 0/3 | 0/3 | 0/3 | 0/3 | 0/3 | 0/3 |
| ^b^Erythema | 0/3 | 0/3 | 1/3 | 0/3 | 0/3 | 0/3 | 0/3 |
| Anorexia | 0/3 | 0/3 | 0/3 | 0/3 | 0/3 | 0/3 | 0/3 |
| Rectal Temperature | 0/3 | 0/3 | 0/3 | 0/3 | 0/3 | 0/3 | 1/3 |
| Urinalysis | 0/3 | 0/3 | 0/3 | 0/3 | 0/3 | 0/3 | 0/3 |
| ^c^Hematology | 0/3 | 0/3 | 1 /3 | 0/3 | 1/3 | 1/3 | 2/3 |
| Fibrinogen | 0/3 | 0/3 | 0/3 | 1/3 | 1/3 | 2/3 | 3/3 |
| C-Reactive Protein | 0/3 | 0/3 | 0/3 | 0/3 | 1/3 | 2/3 | 3/3 |
| ^d,e^Serum Chemistry | 1/3 | 1/3 | 0 /3 | 1/3 | 0/3 | 1/3 | 0/3 |

^a^Number of animals with reactions / total number of animal in the vaccine group (N = 3).

^b^The draize dermal irritation scoring system was used for monitoring injection site reactions.

^c^Mild increases of 1.6-2.3 fold in neutrophils were observed 24 hours after boosting.

^d^Mild increases in creatinine kinase (ALT, AST) were observed.

^e^Serum chemistry panel included: albumin, alkaline phosphatase, alanine aminotransferase, aspartate transaminase, total bilirubin, calcium, total cholesterol, creatine kinase, creatinine, C-reactive protein, gamma glutamyltransaminase, glucose, inorganic phosphorus, total protein, triglyceride, blood urea nitrogen, globulin, albumin/globulin ratio, chloride, potassium, and sodium.
