# Supplementary material for: A Synthetic Adjuvant to Enhance and Expand Immune Responses to Influenza Vaccines
Source: PLoS One. 2010 Oct 27;5(10):e13677. doi: 10.1371/journal.pone.0013677 (PMC2965144; doi:10.1371/journal.pone.0013677)
Supplement: Table S2 — (0.04 MB DOC) [file pone.0013677.s003.doc]

**Supplemental Table 2.** Number of NHP with HI titers ≥40 in animals immunized with adjuvanted Fluzone vaccines.

|  | A/H1N1/ Solomon Islands | | A/H3N2/ Wisconsin | | B/Malaysia | |
| --- | --- | --- | --- | --- | --- | --- |
| Vaccines | ^a^Day 30 | ^b^Day 58 | Day 30 | Day 58 | Day 30 | Day 58 |
| Fluzone | ^c^1/3 | 1/3 | 1/3 | 3/3 | 1/3 | 1/3 |
| Fluzone+ SE | 2/3 | 3/3 | 3/3 | 3/3 | 1/3 | 3/3 |
| Fluzone+  GLA-SE (1µg) | 2/3 | 3/3 | 2/3 | 3/3 | 0/3 | 3/3 |
| Fluzone+  GLA-SE (5µg) | 2/3 | 3/3 | 3/3 | 3/3 | 2/3 | 3/3 |
| Fluzone+  GLA-SE (25µg) | 3/3 | 3/3 | 3/3 | 3/3 | 2/3 | 3/3 |
| Fluzone+  GLA-SE (50µg) | 2/3 | 3/3 | 3/3 | 3/3 | 1/3 | 3/3 |

Titers determined after ^a^one and ^b^two immunizations.

^c^Number of animals with ≥40 titers / total number of animals in the group (N = 3).
