# Supplementary material for: A Synthetic Adjuvant to Enhance and Expand Immune Responses to Influenza Vaccines
Source: PLoS One. 2010 Oct 27;5(10):e13677. doi: 10.1371/journal.pone.0013677 (PMC2965144; doi:10.1371/journal.pone.0013677)
Supplement: Table S3 — (0.04 MB DOC) [file pone.0013677.s004.doc]

**Supplemental Table 3.** Cross-reactive HI titers in NHP immunized with Fluzone ± adjuvants.

|  | A/H1N1 | | | A/H3N2 |
| --- | --- | --- | --- | --- |
| Vaccines | Brisbane | New Caledonia | Beijing | Uruguay |
| Fluzone | ^a^0/3 | 0/3 | 0/3 | 0/3 |
| Fluzone+ SE | 0/3 | 0/3 | 0/3 | 1/3 |
| Fluzone+  GLA-SE (1µg ) | 0/3 | 0/3 | 0/3 | 2/3 |
| Fluzone+  GLA-SE (5µg ) | 3/3 | 3/3 | 1/3 | 3/3 |
| Fluzone+  GLA-SE (25µg ) | 3/3 | 3/3 | 0/3 | 3/3 |
| Fluzone+  GLA-SE (50µg ) | 3/3 | 3/3 | 1/3 | 3/3 |

^a^Number of animals with ≥40 HI titers cross-reactive to drifted H1N1 and

H3N2 strains / total number of animals in the group (N = 3) on day 58 after

two vaccine injections.
